# Sequential Bearings-Only-Tracking Initiation with Particle Filtering Method

**DOI:** 10.1155/2013/489121

**Published:** 2013-12-25

**Authors:** Bin Liu, Chengpeng Hao

**Affiliations:** ^1^School of Computer Science and Technology, Nanjing University of Posts and Telecommunications, Nanjing 210023, China; ^2^Institute of Acoustics, Chinese Academy of Sciences, Beijing 100190, China

## Abstract

The tracking initiation problem is examined in the context of autonomous bearings-only-tracking (BOT) of a single appearing/disappearing target in the presence of clutter measurements. In general, this problem suffers from a combinatorial explosion in the number of potential tracks resulted from the uncertainty in the linkage between the target and the measurement (a.k.a the data association problem). In addition, the nonlinear measurements lead to a non-Gaussian posterior probability density function (pdf) in the optimal Bayesian sequential estimation framework. The consequence of this nonlinear/non-Gaussian context is the absence of a closed-form solution. This paper models the linkage uncertainty and the nonlinear/non-Gaussian estimation problem jointly with solid Bayesian formalism. A particle filtering (PF) algorithm is derived for estimating the model's parameters in a sequential manner. Numerical results show that the proposed solution provides a significant benefit over the most commonly used methods, IPDA and IMMPDA. The posterior Cramér-Rao bounds are also involved for performance evaluation.

## 1. Introduction

The problem of angle/bearings-only tracking (BOT) has been studied over many years due to its tremendous importance in a variety of practical communication and signal processing applications, such as localization in wireless sensor networks [1], submarine tracking using passive sonar [2], aircraft surveillance using radars [3], and sonar-based robotic navigation [4, 5]. This paper is concerned with radar and sonar tracking. Specifically, the focus of this paper lies in a fundamental problem in many sonar and radar tracking tasks, called track initiation. This problem consists in linking sets of point observations from different time steps to fit a desired model without any *a priori* track estimates. In general, track initiation approaches can be categorized into two schemes: sequential and batch schemes [6–8]. The sequential scheme processes a sequence of measurements received during consecutive radar/sonar scans sequentially one at a time. The sequential methods are widely used in radar and sonar tracking problems. For the batch technique approach, *N* past scans of measurements are treated simultaneously to determine feasible target trajectories (see Figure 2). The batch techniques suffer from a heavy computational load and a slow process, while they find applications in image processing and tracking in heavy clutter background [6]. This paper only concerns sequential techniques.

In the context of radar and sonar applications, the target signal, if present, is often corrupted by additive noise and comes with other measurements unrelated to the target, which originate from thermal noise and various forms of clutter such as terrain and clouds [9]. So the issue of measurement origin uncertainty has to be addressed before tracking. This is called the data association problem and has been addressed in the context of multitarget tracking (MTT) in clutter [10, 11].

The tracking initiation problem considered here has its own challenges not only due to the measurement origin uncertainty, but also the uncertainty in the target presence. In fact, for tracking initiation, it is prerequisite to determine whether the target is present before dealing with data association and state filtering. Moreover, in BOT the measurement function is highly nonlinear, which results in a non-Gaussian posterior probability density function (pdf) in the optimal estimate of the target. The consequence of this nonlinear/non-Gaussian context is the absence of a closed-form solution [12–14]. As BOT initiation involves such interactive problems, that is, the measurement origin uncertainty, the target presence uncertainty, and the nonlinear/non-Gaussian posteriors in optimal estimation, it is therefore much more difficult than any individual-related tasks such as state filtering, target detection, and data association. It is noted that, besides the simulated annealing [15, 16] and the Ants algorithm [17], there are few reports on such BOT initiation problem, not to mention sequential BOT initiation.

This paper proposes an algorithm for sequential BOT initiation. The measurement origin uncertainty, target presence uncertainty, and the nonlinear non-Gaussian factors are handled jointly within a Bayesian sequential estimation framework. Based on such Bayesian formalism, a sequential Monte Carlo (SMC), a.k.a particle filtering (PF), algorithm is derived. Performance of the proposed approach is evaluated via numerical simulations and related methods are involved for performance comparison.

The remainder of this paper is organized as follows.  Section 2 formulates the sequential BOT initiation problem.  Section 3 presents the Bayesian sequential estimation framework for the problem under consideration.  Section 4 derives the PF algorithm under the framework developed above. In Section 5, simulations are performed to evaluate the performance of the proposed algorithm. Finally, Section 6 concludes the paper.

## 2. Problem Formulation

### 2.1. Dynamic Model of the Target

Consider a discrete-time dynamic model

(1) $$\begin{array}{c} x_{k+1}=f_{k}\left(x_{k},v_{k}\right), \end{array}$$

which is used to describe the target movement in a two-dimensional (2D) *x*-*y* plane. *k* denotes the discrete time index, **v**_*k*_ the process noise, and **x**_*k*_ the target state vector. The target state consists of 2D position and velocity elements, which is defined as below:

(2) $$\begin{array}{c} x_{k}={\left[\begin{array}{cccc} x_{k} & {\dot{x}}_{k} & y_{k} & {\dot{y}}_{k} \end{array}\right]}^{T}, \end{array}$$

where (*x*, *y*) and $\left(\dot{x},\dot{y}\right)$ denote the 2D position and velocity, respectively. The superscript *T* denotes matrix transposition. If the movement pattern of the target is *a priori *known, then **f** will have a specific form. For example, if the target moves with a near constant velocity, then **f** can be modeled to be

(3) $$\begin{array}{c} f_{k}\left(x_{k},v_{k}\right)=Fx_{k-1}+v_{k}, \end{array}$$

where $F=\left[\begin{array}{cc} F_{s} & 0\\ 0 & F_{s} \end{array}\right]$,  $F_{s}=\left[\begin{array}{cc} 1 & \text{T}\\ 0 & 1 \end{array}\right]$, and T denotes the sampling period of the measurements. For more details on dynamic models for tracking problems, see [18].

### 2.2. Markov Chain Model for Uncertainty of Target Existence

In tracking initiation problem, the target presence is not a definitive issue either before the track under consideration is confirmed or after it is terminated. To take into account the uncertainty of the target presence, we introduce binary valued variable *λ*_*k*_ ∈ {0,1} and let *λ*_*k*_ = 1 represent the target being present in the surveillance region at time *k*, and *λ*_*k*_ = 0 denotes the opposite. Evolution of *λ* during the surveillance time is modeled by a Markov chain, which is characterized by the following transition probabilities:

(4) $$\begin{array}{c} P_{b}≜P\left\{\lambda_{k}=1\;|\;\lambda_{k-1}=0\right\}, \end{array}$$

(5) $$\begin{array}{c} P_{d}≜P\left\{\lambda_{k}=0\;|\;\lambda_{k-1}=1\right\}, \end{array}$$

and the initial probability of target existence, that is, *p*(*λ*_1_). *P*_*b*_ and *P*_*d*_, have specific physical meanings. *P*_*b*_ is actually the probability of a new target's birth or appearing at a time step and *P*_*d*_ is the probability of the target's death or disappeance. The transitional probability matrix is given by

(6) $$\begin{array}{c} \Pi =\left[\begin{array}{cc} 1-P_{b} & P_{b}\\ P_{d} & 1-P_{d} \end{array}\right], \end{array}$$

where 1 − *P*_*d*_ and 1 − *P*_*b*_ are equivalent to probabilities of target staying alive and remaining absent, respectively. *P*_*b*_, *P*_*d*_, and *p*(*λ*_1_) are assumed *a priori *known.

### 2.3. Measurement Model

Let **Z**_*k*_≜(**z**_1_, **z**_2_,…, **z**_*k*_) denote the measurements received up to and including the time step *k*. Here, **z**_*k*_ represents the measurement received at time *k*. It is composed of only clutters (if the target is absent currently) or clutters along with the target originated measurement element (if the target is present at this time step). Specifically,

(7) $$\begin{array}{c} z_{k}≜\{\begin{array}{cc} \left(\beta_{k},z_{1,k},z_{2,k},\ldots ,z_{m_{k},k}\right), & \text{if}\;\;\lambda_{k}=1\\ \left(z_{1,k},z_{2,k},\ldots ,z_{m_{k},k}\right), & \text{if}\;\;\lambda_{k}=0, \end{array} \end{array}$$

where *β*_*k*_ denotes the target originated measurement, *z*_1,*k*_, *z*_2,*k*_,…, *z*_*m*_*k*_,*k*_ measurement elements due to clutters, and *m*_*k*_ the number of clutters. Note that elements in **z**_*k*_ are not ordinal. For BOT, the relationship between *β*_*k*_ and **x**_*k*_ is specified to be

(8) $$\begin{array}{c} \beta_{k}=\arctan\left(\frac{x_{k}-x_{o,k}}{y_{k}-y_{o,k}}\right)+w_{k}, \end{array}$$

where **w**_*k*_ is the measurement noise with known pdf and is independent of **v**_*k*_ in (1) and (*x*_*o*,*k*_, *y*_*o*,*k*_) the sensor's position.

The measurement elements due to clutters are assumed to be uniformly distributed over the surveillance region, so the pdf *p*_*c*_(·) = 1/*V*, where *V* denotes volume of the surveillance region. The number of clutters per scan, *m*_*k*_, is Poisson distributed with density parameter *M*_*c*_; that is, *m*_*k*_~Poission(*M*_*c*_).

### 2.4. The BOT Initiation Problem

The information on target presence along with the target's dynamic state (if target presence has been confirmed) is of interest here, while the only probe to get the desired information is the received measurements.

**Figure 1:**
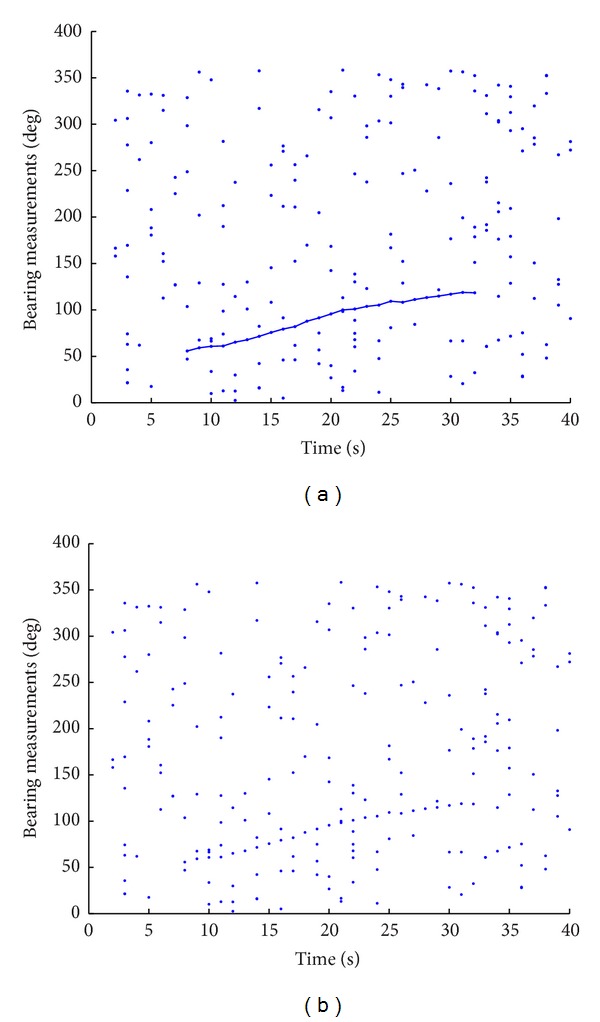
Received BOT measurements versus discrete time index. The left plot indicates target originated measurements by crossing them by a line, while the right plot does not give any indication on the source of each measurement.

**Figure 2:**
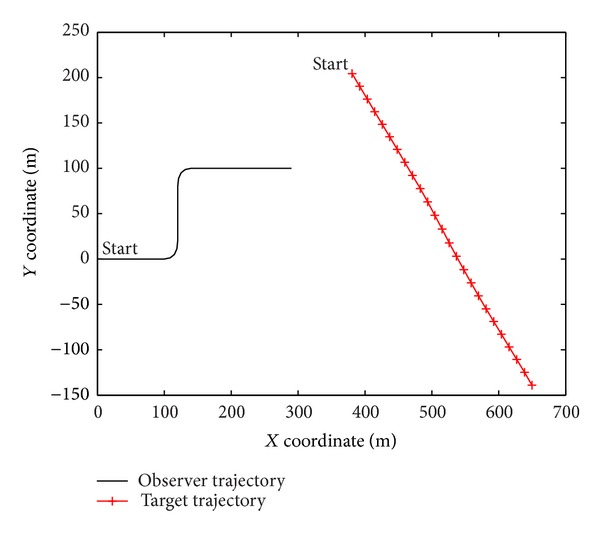
The observer trajectory and the target trajectory in the simulation.

In Figure 1, we give an example showing the measurements generated in a BOT initiation case, where the target exists from the 8th second to the 32nd second. In the upper plot, the target-originated measurements are linked by a series of line segments, while the other points denote measurements generated due to clutters. The bottom plot represents the true scenario encountered in practice where there is no indication on the source of the measurements.

The task of track initiation just consists in taking sets of point observations from different time steps and linking together those observations that fit a desired model without any previous track estimates. As shown in Figure 1, we may draw many different lines to link many different sets of measurements, while there is only one true answer underlying these measurements. In general, such track initiation problem suffers from a combinatorial explosion in the number of potential tracks that must be evaluated.

As indicated by Figure 1, it is reasonable to input the measurements to the tracker algorithm in a batch mode, as latter observations can be used to benefit making decisions at previous time steps. That is why so many batch mode techniques are developed for track initiation, for example, the Hough-transform-based approach [6, 8, 19–21], the multiple-kd tree algorithm [22], and so on.

However, the problem of interest here is different. The focus is how to do BOT initiation in a sequential manner. We formulate this problem as follows. Given *p*(**x**_*k*_, *λ*_*k*_ | **Z**_*k*_), once **z**_*k*+1_ is observed, how to calculate *p*(**x**_*k*+1_, *λ*_*k*+1_ | **Z**_*k*+1_) immediately, *k* = 1,2,…. Actually, *p*(**x**_*k*_, *λ*_*k*_ | **Z**_*k*_) covers all the information about (**x**_*k*_, *λ*_*k*_) that we are able to get based on **Z**_*k*_. It is noted that, provided that sufficient statistics of *p*(**x**_*k*_, *λ*_*k*_ | **Z**_*k*_) can be obtained, there is no need to reload and process **Z**_*k*_ for calculating *p*(**x**_*k*+1_, *λ*_*k*+1_ | **Z**_*k*+1_) upon the arrival of **z**_*k*+1_.

## 3. Sequential Bayesian Estimation Framework for BOT Initiation

Here, the distribution of interest is the posterior *p*(**x**_*k*_, *λ*_*k*_ | **Z**_*k*_). We present a Bayesian estimation framework for computing this posterior distribution sequentially in a recursive manner. The recursion starts from an initial distribution (**x**_0_, *λ*_0_) ~ *p*(**x**_0_, *λ*_0_) | **Z**_0_, where **Z**_0_ is empty, since at the very beginning, there is no measurement element received. The successive posteriors are computed as follows:

(9) p(xk,λk ∣ Zk−1) =∫p(xk,λk ∣ xk−1,λk−1)p(xk−1,λk−1 ∣ Zk−1)   ×d(xk−1,λk−1)p(xk,λk ∣ Zk)∝p(zk ∣ xk,λk)p(xk,λk ∣ Zk−1).

Now let us examine individual parts that constitute the above integrals. First, consider the state transition part:

(10) $$\begin{array}{c} p\left(x_{k},\lambda_{k}\;|\;x_{k-1},\lambda_{k-1}\right)=p\left(x_{k}\;|\;x_{k-1},\lambda_{k-1},\lambda_{k}\right)p\left(\lambda_{k}\;|\;\lambda_{k-1}\right). \end{array}$$

Note that *p*(*λ*_*k*_ | *λ*_*k*−1_) is just determined by the birth/death Markov model (6). If *λ*_*k*_ = 0, the target is absent, so **x**_*k*_ is undefined; otherwise, the pdf of **x**_*k*_ conditional on **x**_*k*−1_ and *λ*_*k*−1_ is given by

(11) $$\begin{array}{c} p\left(x_{k}\;|\;x_{k-1},\lambda_{k-1},\lambda_{k}=1\right)=\{\begin{array}{cc} p\left(x_{k}\;|\;x_{k-1}\right) & \text{for}\;\lambda_{k-1}=1,\\ p_{B}\left(x_{k}\right) & \text{for}\;\lambda_{k-1}=0, \end{array} \end{array}$$

where *p*_*B*_(·) denotes the initial pdf of the target on its appearance.

Now, we consider the calculation of the measurement likelihood. To begin with, introduce variable *n*_*k*_ to denote the number of measurement elements in **z**_*k*_. If the target is absent, that is, *λ*_*k*_ = 0, then *n*_*k*_ just equals the number of measurements due to clutters, that is, *m*_*k*_; otherwise, *n*_*k*_ = *m*_*k*_ + 1. Now, we introduce the association variable *γ*_*k*_. We use *γ*_*k*_ = *i*, *i* ∈ {1,…, *n*_*k*_}, to denote the event that the *i*th element in **z**_*k*_ is target-originated, and the others are generated due to clutters. Additionally, *γ*_*k*_ = 0 denotes that no elements in **z**_*k*_ are target-originated. Now, the measurement likelihood is specified to be

(12) $$\begin{array}{c} p\left(z_{k}\;|\;x_{k},\lambda_{k}\right)=\overset{n_{k}}{\underset{i=0}{\sum }}p\left(z_{k}\;|\;x_{k},\lambda_{k},\gamma_{k}=i\right)p\left(\gamma_{k}=i\;|\;x_{k},\lambda_{k}\right), \end{array}$$

where

(13) p(zk ∣ xk,λk,γk=i) ={p(zi,k ∣ xk)·(pc(·))mk,if    λk=1,  i≠0,(pc(·))nk,if    λk=0,  i=0,0,otherwise,p(γk=i ∣ xk,λk) ={1nk,if  λk=1,  i=1,…,nk,1,if  λk=0,  i=0,0,otherwise.

## 4. Sequential Monte Carlo Implementation

In this section, SMC techniques are developed to approximately implement the Bayesian estimation framework presented in Section 3. The idea is to use the Monte Carlo sampling methodology to approximate the involved distributions by a set of weighted random samples. It is noted that convergence of SMC methods in dealing with nonlinear non-Gaussian Bayesian tracking problem has been proved [23–25].

To begin with, denote {**x**_*k*−1_^*n*^, *λ*_*k*−1_^*n*^, *w*_*k*−1_^*n*^}_*n*=1_^*N*^ as a random measure that approximates the posterior at *k* − 1; namely, *p*(**x**_*k*−1_, *λ*_*k*−1_ | **Z**_*k*−1_). Here, *N* is the sample size; *w*_*k*−1_^*n*^ is the importance weight of the *n*th sample (**x**_*k*−1_^*n*^, *λ*_*k*−1_^*n*^). In the following, an attempt is made to derive an evolution law for the current random set of particles to get a particle approximation to *p*(**x**_*k*_, *λ*_*k*_ | **Z**_*k*_).

First, evolve the existence variable one time step further for each particle based on the birth/death Markov model (6). For example, if *λ*_*k*−1_^*n*^ = 0, then the probabilities of *λ*_*k*_^*n*^ = 1 and *λ*_*k*_^*n*^ = 0 are *P*_*b*_ and 1 − *P*_*b*_, respectively, according to (4) and (6).

Next, we show how to generate state vector value **x**_*k*_ for each particle according to (10) and (11). Let us focus on particles that are associated with positive *λ*_*k*_ values. They are active particles at time step *k*. These particles can be categorized into two classes:

- *newborn particles*: this set of particles is characterized by the transition from *λ*_*k*−1_^*n*^ = 0 to *λ*_*k*_^*n*^ = 1;
- *staying alive particles*: this is a set of particles that continues to stay active with *λ*_*k*−1_^*n*^ = 1 and *λ*_*k*_^*n*^ = 1.

According to (11), we draw state vector sample values **x**_*k*_ for the *newborn particles* from a given initial pdf *p*_*B*_(·) and draw state vector sample values for the *staying alive particles* from the dynamic transition prior density, which are determined by the target dynamic model (1). For the rest of particles that are associated with *λ*_*k*_ = 0, just keep their state vector values undefined, since they are totally useless in the algorithm.

Now we see, for each particle, say the *n*th, its associated state vector value **x**_*k*_^*n*^ and existence variable value *λ*_*k*_^*n*^ are obtained, while the importance weight *w*_*k*_^*n*^ needs to be updated when the new measurement **z**_*k*_ arrives at time step *k*. We update the importance weights according to the solid Bayesian formalism given by (9). Given **x**_*k*_^*n*^ and *λ*_*k*_^*n*^, the (unnormalized) importance weight ${\tilde{w}}_{k}^{n}$ is just equivalent to the likelihood of **z**_*k*_. It is calculated according to (12). A normalization step is processed as follows:

(14) $$\begin{array}{c} w_{k}^{n}=\frac{{\tilde{w}}_{k}^{n}}{\sum_{k=1}^{N}{\tilde{w}}_{k}^{n}}, \end{array}$$

to make sure that the summation of the importance weights equals 1. Then, the posterior probability of target existence, that is, *p*(*λ*_*k*_ = 1 | **Z**_*k*_), is computed as below

(15) $$\begin{array}{c} P_{k}=\overset{N}{\underset{n=1}{\sum }}\lambda_{k}^{n}w_{k}^{n}, \end{array}$$

which satisfies 0 ≤ *P*_*k*_ ≤ 1. The target is declared to be present if *P*_*k*_ is bigger than a given threshold, 0.6 is used in our simulation test. If the target is determined to be present, its state is estimated as follows:

(16) $$\begin{array}{c} {\hat{x}}_{k\;|\;k}=\frac{\left(\sum_{n\in \{1,\ldots ,N\}:\lambda_{k}^{n}=1}^{}x_{k}^{n}w_{k}^{n}\right)}{\left(\sum_{n\in \{1,\ldots ,N\}:\lambda_{k}^{n}=1}^{}w_{k}^{n}\right)}. \end{array}$$

To avoid the problem of degeneracy of SMC algorithm, that is, avoiding the situation that all but one of the importance weights are close to zero, a resampling procedure is performed if the effective number of particles is less than a given threshold *N*_thr_. An estimate of the effective number of particles is computed as follows [26]:

(17) $$\begin{array}{c} {\hat{N}}_{\text{eff}}=\frac{1}{\sum_{n=1}^{N}{\left(w_{k}^{n}\right)}^{2}}. \end{array}$$

The resampling procedure can be summarized as follows: draw particles from the current particle set with probabilities proportional to their weights and then replace the current particle set with this new one. For more discussions on resampling methods in context of PF, refer to [23, 27, 28]. The above SMC algorithm is initialized at *k* = 1 by drawing samples *λ*_1_^*n*^, *n* = 1,…, *N* in accordance with the initial target existence probability *μ*_1_. The initialization of the active particles' state vector is the same as for the *newborn particles* described above.

## 5. Simulation Study

In this section, the performance of the proposed PF algorithm for sequential BOT initiation is evaluated via simulations. The estimation accuracy of the proposed algorithm is compared to the Posterior Cramér-Rao lower bounds (CRLB) [29]. Two generally accepted approaches for sequential track initiation, namely, integrated probabilistic data association (IPDA) [9] and integrated multiple model probabilistic data association (IMMPDA) [30, 31], are also involved for performance comparison.

### 5.1. Simulation Setting

The scenario to be investigated involves BOT of a target from an observer, as shown in Figure 1. The observer travels at a fixed speed of 10 m/s and executes 2 maneuvers during the period under surveillance. The scanning process lasts 40 seconds in total. During the first 8 seconds, the target does not emit any energy outward, so there is no target-originated elements in the received measurements. During the period from the 8th to the 32nd second, the target moves and emits energies outward, so the received measurements include the target-originated elements and the tracker algorithm is expected to be able to detect the target. After the 32nd seconds, generation of target-originated elements is terminated in the received measurements. This setting is used to test whether a tracker algorithm under examination can terminate the track in time. The target motion is simulated according to (3) subjected to an amount of process noise with *q*_*s*_ = 0.1. The target's 2D position and velocity are initialized to be (380,200) and (12.25, −12.25), respectively. The other parameters used in this simulation are listed in Table 1. An example show of the received measurements versus discrete time index is presented in Figure 1.

### 5.2. Performance Comparison on Target Detection

**Figure 3:**
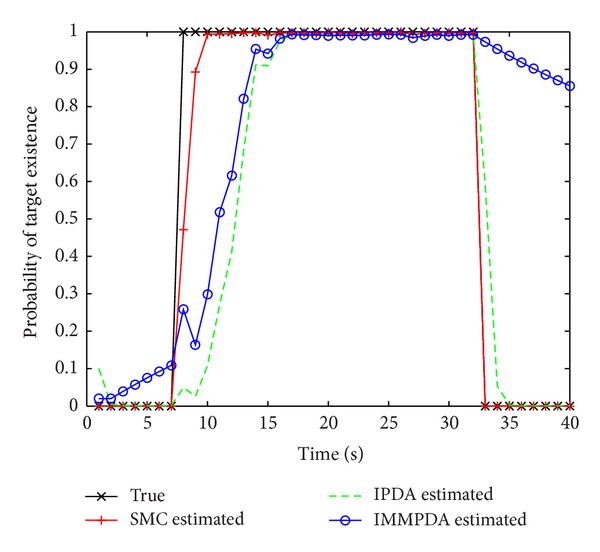
The true and the estimated probabilities of target existence versus time index.

Detection performance of the involved algorithm is investigated via Monte Carlo simulations with 100 independent runs of each algorithm. The outputted probabilities of target existence at each time step are averaged and then plotted in Figure 3. As is shown, the proposed SMC algorithm beats both IPDA and IMMPDA remarkably in the rate of convergence.

Quantitative comparison is also conducted via hypothesis testing. Hypotheses *H*_0_ and *H*_1_ are defined to represent the events “target being absent” and “target being present,” respectively. At each time step *k*, four possible cases may happen for each algorithm:

1. *H*_0_ is true and the algorithm chooses *H*_0_,
2. *H*_0_ is true and the algorithm chooses *H*_1_,
3. *H*_1_ is true and the algorithm chooses *H*_0_,
4. *H*_1_ is true and the algorithm chooses *H*_1_.

We select probability of false alarm, denoted by *P* ^FA^, and probability of detection, denoted by *P* ^*D*^, as the performance metrics. Let *C*_*i*_ count the number of times of case *i* appearance in the simulation test, *i* ∈ {1,2, 3,4}. Then *P* ^FA^ = *C*_2_/(*C*_1_ + *C*_2_) and *P* ^*D*^ = *C*_4_/(*C*_3_ + *C*_4_). So a good algorithm is expected to yield bigger *P* ^*D*^ and smaller *P* ^FA^. *P* ^*D*^ and *P* ^FA^ are calculated over 100 independent runs of each algorithm and the final result is listed in Table 2. It is demonstrated again that the proposed SMC algorithm is superior to IPDA and IMMPDA in detection performance.

### 5.3. Examination on Estimation Accuracy

The posterior Cramer-Rao lower bounds (CRLB) [29] are used to indicate the best estimation accuracy an algorithm can achieve. The performance metric, namely, the root-mean square (RMS) position error is defined as follows:

(18) $$\begin{array}{c} \text{RM}{\text{S}}_{k}=\sqrt{\frac{\sum_{i=1}^{M}{\left({\hat{x}}_{k}^{i}-x_{k}\right)}^{2}+{\left({\hat{y}}_{k}^{i}-y_{k}\right)}^{2}}{M}}, \end{array}$$

where (*x*_*k*_, *y*_*k*_) denotes the target's 2D position at time step *k* in the simulations, $\left({\hat{x}}_{k}^{i},{\hat{y}}_{k}^{i}\right)$ the estimation of (*x*_*k*_, *y*_*k*_) outputted in the *i*th test of the algorithm under consideration, and *M* the number of independent tests under consideration. Define *J*_*x*,*k*_^−1^, *J*_*y*,*k*_^−1^ as the 2D position elements of the inverse information matrix for the problem at hand; the corresponding CRLB for the metric given by (18) is calculated as below:

(19) $$\begin{array}{c} \text{CRLB}\left(\text{RM}{\text{S}}_{k}\right)=\sqrt{J_{x,k}^{-1}+J_{y,k}^{-1}}. \end{array}$$

**Figure 4:**
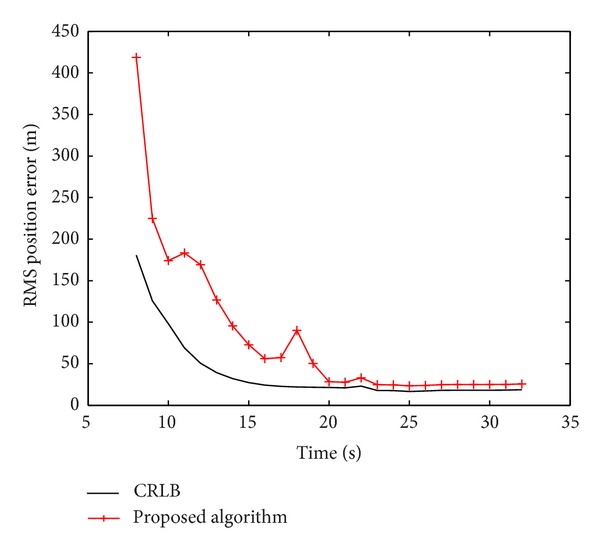
RMS position error in comparison with the posterior CRLB.

Here, we only consider the scanning time interval between the 8th and the 32nd time steps, when the target is truly present and the proposed algorithm successfully detects it.  Figure 4 shows the RMS position error of the proposed algorithm in comparison with the calculated CRLB.

## 6. Conclusions

This paper addresses the problem of sequential BOT initiation in the context of sonar and radar applications. A sequential Bayesian estimation framework is developed for this problem. This theoretical framework addresses measurement origin uncertainty and target existence uncertainty jointly via solid Bayesian formalism. An SMC approximate algorithm is derived under this framework and its performance is evaluated via simulations. It is shown that the proposed algorithm provides a remarkable performance improvement in target detection, compared with the commonly used PDA based methods. The proposed algorithm also gives accurate estimation of the target's state, as indicated by a comparison with the posterior CRLB.

## Figures and Tables

**Table 1:** Parameter setting for simulation.

Symbol	Quantity	Value
*T*	Measurement sampling period	1 s
*σ*	Standard error of measurement noise	1 degree
*M*_*c*_	Expected number of clutters per scan	5
*N*	Sample size used in SMC	3000

**Table 2:** Detection performance comparison.

Algorithms	*P* ^FA^	*P* ^*D*^
The proposed SMC algorithm	0.67%	96%
IPDA	4%	81.2%
IMMPDA	53.33%	86%
